# Factors Affecting General Practitioners’ Readiness to Accept and Use an Electronic Health Record System in the Republic of North Macedonia: A National Survey of General Practitioners

**DOI:** 10.2196/21109

**Published:** 2021-04-05

**Authors:** Tomi Dimitrovski, Peter A Bath, Panayiotis Ketikidis, Lambros Lazuras

**Affiliations:** 1 CITY College University of York Europe Campus Thessaloniki Greece; 2 South-East European Research Centre Thessaloniki Greece; 3 Information School University of Sheffield Sheffield United Kingdom; 4 School of Health and Related Research University of Sheffield Sheffield United Kingdom; 5 Department of Psychology, Sociology & Politics Sheffield Hallam University Sheffield United Kingdom

**Keywords:** general practitioner, eHealth, technology acceptance, electronic health record

## Abstract

**Background:**

Electronic health records (EHRs) represent an important aspect of digital health care, and to promote their use further, we need to better understand the drivers of their acceptance among health care professionals. EHRs are not simple computer applications; they should be considered as a highly integrated set of systems. Technology acceptance theories can be used to better understand users’ intentions to use EHRs. It is recommended to assess factors that determine the future acceptance of a system before it is implemented.

**Objective:**

This study uses a modified version of the Unified Theory of Acceptance and Use of Technology with the aim of examining the factors associated with intentions to use an EHR application among general practitioners (GPs) in the Republic of North Macedonia, a country that has been underrepresented in extant literature. More specifically, this study aims to assess the role of technology acceptance predictors such as performance expectancy, effort expectancy, social influence, facilitating conditions, job relevance, descriptive norms, and satisfaction with existing eHealth systems already implemented in the country.

**Methods:**

A web-based invitation was sent to 1174 GPs, of whom 458 completed the questionnaire (response rate=40.2%). The research instrument assessed performance expectancy, effort expectancy, facilitating conditions, and social influence in relation to the GPs’ intentions to use future EHR systems. Job relevance, descriptive norms, satisfaction with currently used eHealth systems in the country, and computer/internet use were also measured.

**Results:**

Hierarchical linear regression analysis showed that effort expectancy, descriptive norms, social influence, facilitating conditions, and job relevance were significantly associated with intentions to use the future EHR system, but performance expectance was not. Multiple mediation modeling analyses further showed that social influence (*z*=2.64; *P*<.001), facilitating conditions (*z*=4.54; *P*<.001), descriptive norms (*z*=4.91; *P*<.001), and effort expectancy (*z*=5.81; *P*=.008) mediated the association between job relevance and intentions. Finally, moderated regression analysis showed that the association between social influence and usage intention was significantly moderated (*P*=.02) by experience (B_experience×social influence_=.005; 95% CI 0.001 to 0.010; *β*=.080). In addition, the association between social influence and intentions was significantly moderated (*P*=.02) by age (B_age×social influence_=.005; 95% CI 0.001 to 0.010; *β*=.077).

**Conclusions:**

Expectations of less effort in using EHRs and perceptions on supportive infrastructures for enabling EHR use were significantly associated with the greater acceptance of EHRs among GPs. Social norms were also associated with intentions, even more so among older GPs and those with less work experience. The theoretical and practical implications of these findings are also discussed.

## Introduction

### Background

The development and implementation of eHealth and digital technologies in health care have become widespread in recent decades. However, there have been numerous failures in eHealth systems because of the lack of adoption and use of these technologies and systems by health care professionals and other staff in health care systems [1,2]. The underutilization of digital technology in health care settings is evident, although the reasons for this are unclear. The low acceptance of new technologies in health care settings remains to be a challenge for health service management and researchers [1,3,4]. Therefore, it is important to gain a better understanding of the processes underlying health care professionals’ acceptance of novel health care technologies and systems [5-7].

Electronic health record (EHR) systems are an essential part of information and communication technologies (ICTs) within health care settings and organizations. In primary health care, EHR systems have been developed to support the storage, retrieval, and use of patient data over the life course of a patient by general practitioners (GPs) and other health care professionals in primary care.

### Technology Acceptance

**Figure 1:**
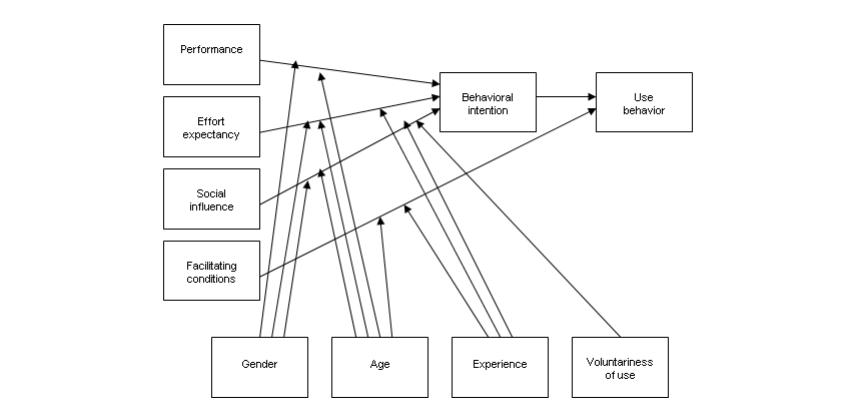
The Unified Theory of Acceptance and Use of Technology.

Different theories have been developed to assess the factors that influence the adoption and use of ICTs in health care, including the Unified Theory of Acceptance and Use of Technology (UTAUT; Figure 1) [8], which seeks to understand the effect of various factors on users’ intentions to use a new system, as well as their actual use of the system. The 4 basic technology acceptance constructs within the UTAUT are performance expectancy, effort expectancy, social influence, and facilitating conditions. Performance expectancy assesses an individual’s anticipation of improved performance resulting from the use of new technologies. Effort expectancy represents the end users’ perceptions of the ease of using new ICTs (ie, how much effort will be required by them to use the new system). Social influence measures the subjective social norms of end users and represents referent others’ endorsement of using the technology in question and the perceived prevalence of the utilization of the technology in referent groups. Facilitating conditions represent the degree to which end users perceive that there will be organizational and technical support for the efficient and easy use of the technology [8]. The original UTAUT model has 4 potential moderators: gender, age, experience, and voluntariness. This means that the association between the UTAUT constructs and usage intentions may be stronger or weaker, depending on the values of the moderator constructs (eg, the association between performance expectancy and intentions to use the technology may be stronger among individuals with more vs less experience in using the technology) [8].

Although the application of early technology acceptance models in health care settings started in the late 1990s [9,10], there is still limited empirical research on technology acceptance in EHR systems. Research on technology acceptance in health care has suggested that performance expectancy is the strongest and most important predictor of intentions to use EHR systems [8,11-14]. Effort expectancy has been shown to be a significant predictor of intentions to use EHR systems [13-17] in a smaller number of studies, and social influence and facilitating conditions have rarely been investigated [14]. A number of additional technology acceptance constructs have been applied in several studies in health care settings, including health information technology experience [7,18], computer knowledge [19], job relevance [20], and the self-assessment of computer use at home [19]. For this research, these constructs can be considered in their original or modified forms. The UTAUT model was applied in mandatory health care settings (where EHR use is compulsory) in the relevant literature [13,14,20].

### This Study

This study is a part of a PhD thesis and is published for the first time in a journal. A national EHR system has been proposed for the Republic of North Macedonia, and the aim of this study is to examine the factors that influence the adoption of such a system among GPs within the country. All GPs in the country worked in private settings, but they had active contracts with the National Health Insurance Fund and were obliged to follow the work instructions proposed by the fund. The proposed EHR system was not implemented in the country when this research was conducted. The technology acceptance assessment was conducted before the implementation of the EHR system in the country with the aim of identifying the factors that determine intentions for future use. However, the “Health Smart Card” system (a smart card access to basic patient personal data and health insurance) and the “My term system” (a web-based scheduling system) were implemented in the country at the time when this research was conducted.

The main objective of this research is to assess the readiness of GPs in the country for the future acceptance of EHR systems. Other objectives are to address the role of the basic predictors of the original UTAUT model on EHR use; to assess the effect of other technology acceptance predictors such as job relevance, descriptive norm, and satisfaction (with existing health ICT systems already implemented in the country); and to identify the moderating effect of basic moderation variables such as age, gender, and previous work experience.

Adding new technology acceptance constructs to the basic UTAUT model was an opportunity to develop a better understanding of the factors influencing the use of ICTs in a large sample of GPs within a country. However, some technology acceptance constructs, such as descriptive norm [21], computer use, internet use, and use of other technology [22-26] were derived and modified from the referent literature studies on technology acceptance and were identified as useful for this research. Descriptive norms can be regarded as a measure of the potential use of EHRs by colleagues.

The following hypotheses were developed:

- H1: the original UTAUT constructs (ie, performance expectancy, effort expectancy, social influence, and facilitating conditions) will be associated with intentions to use the EHR system in the future.
- H2: other technology acceptance constructs—job relevance, satisfaction, and the use of other technology—will be indirectly associated with intentions to use the EHR system in the future through the effects of performance expectancy.
- H3: the association between the basic UTAUT constructs and intentions to use the future EHR system will be moderated by age, gender, and previous work experience (moderation effect according to the UTAUT model).
- H4: descriptive norms will be significantly associated with intentions to use the EHR system in the future, over and above the effects of other predictor constructs.

The assessment of the hypotheses identifies the effects of technology acceptance variables on user intentions. Therefore, this research aims to establish the most important technology acceptance predictors for future EHR systems among GPs in the country.

## Methods

### Recruitment

The target population was the GPs in the country; all GPs who had contracts with the National Health Insurance Fund were included in the study. Participants’ email addresses were provided by the National Health Insurance Fund List. According to the list, there were 1631 active GPs in the country at the time of the study, with 1174 active email addresses of GPs registered in the list. A web-based survey was created on the SharePoint (TM) platform, and an invitation email was sent to all email addresses. General information on the future EHR system is included in a short introduction to the survey. The email was sent on July 1, 2014, followed by 2 reminder emails on July 15, 2014, and August 1, 2014. However, 35 emails were returned, as they did not reach valid email addresses.

### Research Instrument

The original UTAUT model was modified with other technology acceptance extensions for this study. The following technology acceptance items were added to the questionnaire: job relevance [11], descriptive norm (ie, estimated prevalence of EHR use by colleagues in the future) [21,22], current use of other technology for professional or leisure purposes [23], and satisfaction with existing eHealth systems that are currently used in the country. A (user) satisfaction item was developed to assess the GPs’ satisfaction with the currently used ICT systems in health care in the country (the “Health Smart Card” system and the “My term system”). The purpose of including this item was to assess the association of user satisfaction with existing health care ICT systems with the intention of using the future EHR. Job relevance was added to the current research model, as its effectiveness was established in a previous study conducted by researchers [22].

Performance expectancy [8,15] was measured by using 5 questions for assessing aspects of participants’ beliefs about the usefulness of future EHR systems. Effort expectancy [8,11,12,15] was measured by using 8 items for assessing aspects of the ease of use of the future EHR system. Facilitating conditions [8] were measured with 4 items for assessing the degree to which participants believed that organizational infrastructure would support their use of the future EHR system. Social influence [8,12,15] was measured with the mean scores of 3 items for assessing how a participant perceived other colleagues’ beliefs about whether they should use the future EHR system. The descriptive norm [21] variable was measured with a single item that asked participants to estimate how many of their colleagues would use the proposed EHR system if it was implemented. Usage intentions [11,12] were measured by using 4 items for assessing participants’ willingness to use the future EHR system. The job relevance [11,20] of the future EHR system to the GP’s job was measured with 2 items that reflected greater perceived job relevance of the future EHR system to their work tasks. A 5-item measure was adapted from previous research [23-25] to assess the relationship between current computer and internet use for GPs’ professional and personal needs and the current use of other technology with the intention to use the EHR system. Satisfaction with the current system was measured as a possible technology acceptance construct by using 5 questions for measuring participants’ satisfaction with the currently used eHealth systems in the country (“My Term” and “Health Smart Card”).

Questions relating to the 3 UTAUT moderators (ie, gender, age, previous work experience [8]) were also included in the questionnaire. Participants were asked to state their gender, age, and years of work experience in the current service. The voluntariness of use [8] was excluded from the questionnaire because the use of the future EHR in the country will be mandatory, so this question was redundant. The questionnaire was first developed in English and then translated into the Macedonian language using the translation back-translation method [27]. The original questionnaire and technology acceptance constructs used in the research are available from the authors on request [28].

Various approaches such as descriptive statistics, two-tailed independent sample *t* tests, Spearman rank correlations, internal consistency reliability (Cronbach α), hierarchical linear regression, moderated regression analyses, and mediation analyses were applied to analyze the collected data.

### Research Ethics

Research ethics approval was obtained in accordance with the Research Ethics Policy of the University of Sheffield before commencement of the study [29]. The questionnaire was designed to avoid collecting any of the GPs’ personal information. Participants were informed that they could voluntarily participate in the study.

## Results

### Response Rate

A total of 458 completed questionnaires were eligible for analysis, yielding a response rate of 40.2%. The age of the respondents who took part in the study ranged from 24 to 65 years (mean 44.15, SD 11.41). Two-thirds of the participants in the study (303/458, 66.2%) were females and one-third (155/458, 33.8%) were males. The work experience of the participants ranged from <1 year to 38 years of experience (mean 15.45, SD 10.40).

### Reliability

The internal consistency reliability of the technology acceptance constructs used in the questionnaire was assessed using Cronbach α [19,30]. The internal consistency reliability of the measures used in the study ranged from 0.69 to 0.94, suggesting that the measures we used were reliable (Table 1).

**Table 1:** Spearman rank correlations.

Variable			Performance expectancy		Effort expectancy		Facilitating conditions		Job relevance		Social influence		Satisfaction		Descriptive norm		Intention
**Performance expectancy**																	
	*R*	N/A^a^		0.71		0.56		0.66		0.65		0.58		0.61		0.59	
	*P* value	N/A		<.001		<.001		<.001		<.001		<.001		<.001		<.001	
**Effort expectancy**																	
	*R*	N/A		N/A		0.68		0.69		0.66		0.61		0.57		0.68	
	*P* value	N/A		N/A		<.001		.04		<.001		<.001		<.001		<.005	
**Facilitating conditions**																	
	*R*	N/A		N/A		N/A		0.61		0.59		0.58		0.63		0.62	
	*P* value	N/A		N/A		N/A		<.001		<.001		<.001		<.001		<.001	
**Job relevance**																	
	*R*	N/A		N/A		N/A		N/A		0.65		0.55		0.59		0.62	
	*P* value	N/A		N/A		N/A		N/A		<.001		<.001		<.001		<.005	
**Social influence**																	
	*R*	N/A		N/A		N/A		N/A		N/A		0.58		0.68		0.63	
	*P* value	N/A		N/A		N/A		N/A		N/A		<.04		<.001		<.001	
**Satisfaction**																	
	*R*	N/A		N/A		N/A		N/A		N/A		N/A		0.56		0.52	
	*P* value	N/A		N/A		N/A		N/A		N/A		N/A		<.001		<.001	
**Descriptive norm**																	
	*R*	N/A		N/A		N/A		N/A		N/A		N/A		N/A		0.58	
	*P* value	N/A		N/A		N/A		N/A		N/A		N/A		N/A		<.001	
**Intention**																	
	*R*	N/A		N/A		N/A		N/A		N/A		N/A		N/A		N/A	
	*P* value	N/A		N/A		N/A		N/A		N/A		N/A		N/A		N/A	
Mean (SD)			3.95 (1.14)		3.82 (0.87)		4.04 (0.86)		3.87 (1.04)		3.73 (1.10)		3.40 (1.09)		3.96 (1.11)		4.41 (0.91)
Cronbach α			.91		.88		.74		.69		.93		.88		.85		.94

^a^N/A: not applicable.

### Bivariate Correlations

Bivariate correlations were estimated using Spearman rank-order correlation coefficients before the regression analyses. Table 1 presents the Spearman rank correlations.

The Spearman correlation showed that usage intention (the main outcome, ie, the dependent variable of this research) correlated significantly and positively with all the technology acceptance constructs (*R* coefficients=0.52-0.71) included in the study.

### Descriptive Statistics

The participants in this research reported a high performance expectancy from the EHR system. They expressed a positive performance expectancy of over 50% for the system. A small minority (between 10% and 15%) was not favored. Around 18%-24% of the participants were neutral. Respondents also reported high effort expectancy from the system. They reported a positive effort expectancy of over 50% from future EHRs. A small minority (between 7% and 15%) appeared to have a negative attitude, and the neutral responses were higher (between 22% and 31%). Participants reported more than 50% positive agreement with statements on social influence constructs. A smaller minority (between 11% and 13%) reported that they did not agree with the statement, and a consistent proportion (between 30% and 33%) of participants were neutral. Participants reported that facilitating conditions are important for future use of the system. They reported over 50% positive agreement with the statements. A smaller minority of participants (between 6% and 18%) appeared not to be in favor, and 14% to 25% of respondents were neutral. Participants reported over 50% positive agreement with intention statements. A smaller minority (between 4% and 5%) appeared to have low intentions, and between 11% and 14% gave neutral scores on intentions for future use.

### Gender Differences in Technology Acceptance Constructs

Independent sample *t* tests were used to assess gender differences with respect to the technology acceptance constructs. The results indicated that only significant differences were identified in performance expectancy (*t*_456_=2.01; *P*=.04), wherein male GPs reported significantly higher scores (mean 4.10, SD 0.08) than their female colleagues (mean 3.87, SD 1.17).

### Predicting Intentions to Use the EHR System in the Future

Hierarchical linear regression was used to assess the multivariate association between intentions to use the EHR system and UTAUT constructs. The analysis was completed in 2 steps to differentially assess the effects of demographic and information technology use/work-related constructs (entered in the first stage of the analysis) and the effects of technology acceptance constructs (the second step of the analysis). The overall model predicted (*R*^2^) 65.4% of the variance in intention to use the future EHR system *F*_445_=106.77; *P*<.001. In the first step of the analysis, only the use of other technology variables (*β*=−.146; *P*<.001) predicted intention to use the future EHR system. In the second step of the analysis, the addition of the UTAUT constructs significantly increased the predicted variance in intention to use the future EHR system by 63.2%. The significant predictors of intention to use the EHR system at the final step of the analysis included facilitating conditions (*β*=.232; *P*<.001), effort expectancy (*β*=.217; *P*<.001), descriptive norms (*β*=.198, *P*<.001), job relevance (*β*=.172; *P*<.001), and social influence (*β*=.108; *P*=.04). The results of the hierarchical regression analysis are presented in Table 2.

**Table 2:** Predictors of intentions to use the electronic health record system.

Steps: independent constructs			95% CI for unstandardized *β* weights (B)		Standard *β*		Adjusted *R*^2^			*P* value	
**Step 1**								1.8			
	Age (years)	0.007 to 0.021		.091					.31		
	Gender	0.212 to 0.157		.014					.76		
	Work experience	0.022 to 0.007		.092					.29		
	Computer use (years)	0.016 to 0.019		.010					.38		
	Use of other technology	0.593 to 0.110		−.146					.004		
	Use of internet for personal	0.055 to 0.160		.050					.33		
	Use of internet for work	0.089 to 0.142		.021					.65		
**Step 2**								65.4			
	Age (years)	0.014 to 0.004		.062					.25		
	Gender	0.016 to 0.206		.049					.09		
	Work experience	0.015 to 0.003		.049					.34		
	Computer use (years)	0.011 to 0.011		.001					.90		
	Use of other technology	0.144 to 0.151		.001					.96		
	Use of internet for personal	0.060 to 0.069		.004					.89		
	Use of internet for work	0.188 to 0.048		.096					<.001		
	Performance expectancy	0.012 to 0.135		.076					.10		
	Effort expectancy	0.119 to 0.335		.217					<.001		
	Facilitating conditions	0.157 to 0.336		.232					<.001		
	Job relevance	0.070 to 0.232		.172					<.001		
	Social influence	0.016 to 0.162		.108					.01		
	Satisfaction	−0.063 to 0.064		.001					.98		
	Descriptive norm	0.135 to 0.282		.198					<.001		

### Indirect Effects of Job Relevance on Usage Intentions

We used a multiple mediation methodology [31] to assess the indirect effect of job relevance on usage intentions, after controlling for the potential mediation effects of the UTAUT constructs. Bootstrapping and bias-corrected confidence intervals were used to assess the total and indirect effects of the independent variable X (job relevance) on the dependent variable Y (usage intentions), through the effects of multiple mediators, Ms (effort expectancy; social influence, descriptive norm; and facilitating conditions). For the analysis, we used the SPSS Macro Indirect 30 with 1000 resamples and 95% CIs, and the Sobel test (*z*) was used to enable effect size comparisons between the mediators [31].

The mediation analysis showed that the association between job relevance and intentions was mediated by effort expectancy (*z*=5.81; *P*<.001), social influence (*z*=2.64; *P*=.008), descriptive norms (*z*=4.91; *P*<.001), and facilitating conditions (*z*=4.54; *P*<.001). The mediation effect of effort expectancy was significantly higher (*P*=.02) than the effects of social influence and descriptive norms.

### Moderation Effects Between UTAUT Constructs

In total, 8 moderated regression analyses were used to assess the interactive effects of gender, age, and working experience on the relationships between the UTAUT constructs (effort expectancy, social influence, and facilitating conditions) on intentions to use the EHR system. Technology acceptance predictors were mean-centered to avoid multicollinearity [32]. As the direct effect of performance expectancy was nonsignificant, we did not assess the interaction between this variable and gender, age, and experience. An interaction term was computed (independent variable×moderator) for each pair of associations, and each moderated regression analysis was completed in 2 steps. The first step included the main effects of the independent variable and moderator, and the second step included the interaction term. Unstandardized *β* weights (B) and 95% CIs were estimated [32].

The analyses identified only 2 significant moderation effects. Age significantly interacted (*P*=.02) with social influence (B_age×social influence_=.005; 95% CI .001 to .010; *β*=.077), showing that when age was higher, the association between social influence and intentions was stronger (Figure 1). In addition, the relationship between social influence and intention to use the system was significantly moderated (*P*=.02) by experience (B_experience×social influence_=.005; 95% CI 0.001 to 0.010; *β*=.080), showing that among GPs in the early stages of work experience, there was a stronger relationship between the social influence and intentions to use the EHR system.

## Discussion

### Initial Findings

This research identified the significant correlates of technology acceptance predictors for future EHR systems among GPs in the Republic of North Macedonia. On the basis of previous research using the UTAUT in health care settings (8), it was hypothesized that UTAUT constructs (ie, performance expectancy, effort expectancy, facilitating conditions, and social influence) would be associated with intentions to use the EHR system in the future and mediate the relationship of intentions with job relevance, satisfaction with using the eHealth systems in the country, and use of other (non–health care) technology. On the basis of the UTAUT premises [8], it was further hypothesized that the associations between UTAUT constructs and usage intentions would be moderated by age, gender, and previous work experience. Finally, we anticipated that descriptive norms would provide an alternative and useful measure of social norms in the context of UTAUT and health care technologies; therefore, descriptive norms would be significantly associated with usage intentions over and above the effects of other predictors and social norms more specifically.

H1 was accepted, as effort expectancy, social influence, and facilitating conditions constructs were significantly associated with GPs’ intention to use the future EHR system in the multivariate model, which accounted for 65.4% of the variance in intentions. However, although performance expectancy was significantly associated with intentions in the bivariate correlation analysis (Table 1), this association was not significant in the multivariate model. H2 was also supported, as job relevance was significantly and directly associated with usage intentions. H3 was also accepted because age and experience were reported as moderators of the social influence construct. Finally, H4 was accepted as a descriptive norm significantly associated with EHR use intentions.

These findings are in line with previous research [13,14,20], indicating a positive and significant association between effort expectancy and intentions to use health care technology among health care professionals. Although the original UTAUT model [8] posits that performance expectancy is among the strongest predictors of intention to use a system, our study did not support this contention. This is in line with previous research in the Republic of North Macedonia [22]. Facilitating conditions and job relevance were also associated with intentions in this study, and their effect as predictors on EHR intentions had only previously been reported in a limited number of studies [14].

The significant multivariate association between effort expectancy and EHR use intentions corroborates previous research on health care professionals in the Republic of North Macedonia [22]. Taken together, these findings may indicate that GPs in a specific country are not fully aware of the potential benefits of the proposed EHR system and consider perceived effort and supportive infrastructure as more relevant in their decision to use (or not use) such technology. This may explain the nonsignificant multivariate association between performance expectancy and intentions to use the future EHR system. In practical terms, this means that efforts to promote EHR use among GPs in a specific country should address the issue of the ease of using the system (ie, less effort) and the existence of supportive infrastructure, especially among GPs of older age with more years of medical practice experience.

The moderated regression analyses indicated that age and experience moderated the effects of social influence construct on intentions to use the future EHR system. However, no previous studies in these areas have used moderated regression analyses. The mediation analyses showed that the effect of job relevance was mediated by effort expectancy, social influence, descriptive norms, and facilitating conditions. This means that perceiving the use of the EHR system as relevant to GP work can only partially explain the GPs’ decision to use the system. Other, more relevant considerations, such as the perceived effort in using it and the existence of relevant technical support and infrastructure, as well as the perceived use by other GPs, appear to be more prominent considerations in the decision-making process and further explain the association between job relevance and usage intentions. In other words, GPs would be willing to use health care technology that appears relevant to their job, to the extent that this technology is seen as less effortful to use, supported by relevant infrastructure, and endorsed by more colleagues.

### Principal Findings

GPs’ decision to use job-relevant health care technology, such as an EHR system, is multifaceted and based on several considerations. Primarily, the perceived effortless use and the existence of supportive infrastructure appear to be highly relevant to the decision to use the EHR system in question, followed by perceptions of endorsed (and actual) use by colleagues. Taken together, these considerations appear to be more important than the perceived benefits of the EHR system in daily practice.

### Implications for Design and Implementation of EHR Systems

The findings of this study may be useful for policy makers and managers when developing and implementing new ICTs in health care. The contextualized technology acceptance model developed for this study contributes to understanding the drivers of the acceptance of new technology in this country in Southeast Europe. The emphasis on the design and development of future EHRs should be easy to use. The effect of social influence on intentions to use the EHR system may be moderated by the age and experience (moderators) of the GPs.

Managers and policy makers should use workshops and tools that will persuade end users about the ease of use of EHR systems. Future EHR systems should provide effective technical support (facilitating conditions). The influence of key colleagues may facilitate the implementation of EHR systems (social influence).

### Limitations

A quantitative approach was applied in this study, and it is possible that a qualitative approach may have provided a more in-depth explanation of participants’ attitudes. GPs who use ICT less in their professional roles may have been underrepresented in this study, which may have created a response bias in this sample. It is also possible that the views in the research, in relation to readiness to adopt the EHR system, reflect those GPs who were more familiar with using ICT. The response rate of 40.2% is not ideal for generalizing the findings of this research to the whole GP population in the country. Although the research instrument was applied to a large sample of GPs, there was a self-selection bias among respondents. The EHR system in 2020, although planned, was not implemented in the Republic of North Macedonia. However, it is possible that the views of health care professionals, such as performance expectancy and various forms of computer and internet use, have changed over time.

Gender split and other demographic data in the GP population in the country were not available through the National Fund of Health Insurance. The gender distribution in the respondents (2/3 female vs 1/3 male) of this research cannot be compared with the GPs’ gender split. Data for this research were collected in 2014, and there is a time gap with its presentation in this study.

The application of a newer technology acceptance model, such as UTAUT2, was considered a possible limitation. However, the newly added variables to the UTAUT2, such as hedonic motivation (enjoyment derived from using the technology) and price value (trade-off between perceived benefits and monetary costs) were less relevant to the planned EHR (proposed mandatory use of the EHR system reimbursed by the government).

### Comparison With Prior Work

The UTAUT model has been applied in a few studies in health care settings to assess the intentions to use the EHR system. Performance expectancy and effort expectancy were found to be strong predictors, which is different from the findings of this study. The main finding of this study that only effort expectancy (not performance expectancy) was established as a predictor of intention to use the EHR system is different from those in the relevant literature [13,14]. Job relevance was assessed and proved to be a predictor of intention in this study. However, this technology acceptance construct was assessed and established as a predictor of intention among health care professionals in the relevant literature [20]. Social influence, a technology acceptance construct similar to subjective norms, has been more widely used and has been shown to be a behavioral predictor in health care settings in the relevant literature. The findings of this study, where social influence was established as a behavioral predictor, correspond with those described in the literature [14,18,20,33]. However, as performance expectancy was not established as a technology acceptance predictor of intentions in this study, there may be a gap in the awareness of its expected benefits. Therefore, the possible awareness gap may be explored in future research.

### Conclusions

The modified version of the UTAUT applied in this study is a useful tool for researchers to assess attitudes and intentions to use new eHealth systems. The main findings from this study indicated that effort expectancy (not performance expectancy) and facilitating conditions (ie, perceived tech support and supportive infrastructure) were the strongest predictors of intentions for the future use of the EHR system among GPs. Taken together, the main findings of our study suggest that health care technology acceptance can be explained by models, such as the UTAUT model. However, different variables appear to predict intentions to use health care technology in different countries, suggesting that future research may address cultural and contextual influences in health care technology acceptance and that modified versions of the UTAUT may be relevant in different countries.

## Abbreviations

EHR: electronic health record
GP: general practitioner
ICT: information and communication technology
UTAUT: Unified Theory of Acceptance and Use of Technology
